# 全自动进样与馏分收集一体设备的研制

**DOI:** 10.3724/SP.J.1123.2024.12005

**Published:** 2025-07-08

**Authors:** Xinying ZHOU, Weiheng KONG, Dexiu YU, Tangyi LI, Zhou MA, Xin YI, Fenglin WANG, Tong LI

**Affiliations:** 1.依利特（大连）分析仪器有限公司，辽宁 大连 116023; 1. Elite （Dalian） Analytical Instruments Co. ，Ltd. ，Dalian 116023，China; 2.苏州依利特科技有限公司，江苏 苏州 215001; 2. Suzhou Elite Technology Co. ，Ltd. ，Suzhou 215001，China; 3.中国海关科学技术研究中心，北京 100026; 3. Science and Technology Research Center of China Customs，Beijing 100026，China

**Keywords:** 自动进样, 馏分收集, 制备色谱, 设备研制, automatic samples, fraction collection, preparative chromatography, equipment development

## Abstract

制备色谱在制药、化工和食品等领域中扮演着重要的分离与纯化角色。为了提升其分离效率及使用便捷性，研发了一款新型的制备型色谱设备，该设备集成了自动进样与馏分收集功能，旨在满足现代工业对于高效分离和精确收集的需求。本文详细介绍了该设备的设计理念和工作原理。设备通过先进的自动化技术，显著提高了进样速率，并降低了人为操作的误差，使得整个实验过程更加科学和高效。同时，馏分收集系统也经过优化，可以根据不同的应用需求进行灵活调整，确保所收集的目标成分具有更高的纯度与回收率。本文创新地采用高速伺服电机驱动X、Y、Z轴独立运行，使得仪器在设定托盘范围内任意位置完成高速进样和收集动作，实现了样品进样和收集的同步进行；同时利用下位机控制，实现对进样和收集位置的校准，保证进样和收集位置的准确性；此外，本设备可兼容多种规格样品托盘，可以满足1~200 mL/min流量范围内的制备需求。对该设备的重复性和可靠性进行考察，结果表明重复性和可靠性指标均能达到使用要求。利用该设备的进样和收集功能对甜菊苷和莱鲍迪苷A样品进行分离和纯化，该设备显示出优异的性能，包括快速分离、稳定输出和精准收集。结果表明，该设备在实现进样分离的同时，可完成样品收集动作，为相关行业提供了稳定可靠的分离与纯化解决方案。

## Abstract (en)

Preparative chromatography plays an important separation and purification role in the pharmaceutical， chemical， and food industries. New preparative chromatography equipment that integrates automatic sampling and fraction-collecting functions was developed to improve separation efficiency and ease of use， and to meet the separation-efficiency and collection-accuracy needs of modern industry.The design concept and working principles of the device are introduced in detail. The developed equipment significantly improves the injection rate and reduces human operating errors through advanced automation technology， leading to an experimental process that is entirely more scientific and efficient. At the same time， the fraction-collection system was optimized such that it can flexibly adjust to various application requirements， thereby ensuring the highest purities and recoveries of the collected target ingredients. This innovative equipment uses a high-speed servo motor to independently drive the X-， Y-， and Z-axes of the instrument tray in order to realize high-speed sampling and collection actions at any position within the scope of the set tray， while synchronously sampling and collecting. A control circuit was used to calibrate the sampling and collection positions to ensure accuracy and that the device is compatible with a variety of sample-tray specifications to meet the preparation requirements of a wide 1–200 mL/min flow range. The developed equipment was used to separate and purify samples of stevioside and rebaudioside A， and exhibited excellent performance， including fast separation， stable output， and accurate collection. The equipment is capable of realizing synchronous sample-collection functions， thereby providing a stable and reliable separation and purification option for a wide range of industries.

随着我国经济发展和科技创新，我们对科学仪器的研发和应用有着越来越高的要求^［1-3］^。近几年我国在中草药、生物制品和食品研究等领域发展迅速，特别是在植物中有效成分的提纯、生物多肽小分子的纯化等方面，对基于色谱原理的制备提取应用越来越广泛^［4-6］^，客户需求也越来越多样化。因此迫切需要研制一款操作简单、成本低廉、省时省力的制备型色谱设备。

目前制备型色谱设备进样方式有手动进样和自动进样两种，相比手动进样，自动进样因其精度高、重复性好、效率高等特点被广泛应用。特别是在色谱和基于色谱原理的制备领域中，自动进样器可精准地移取指定体积的样品，并自动进入系统中，协助系统实现快速分离。馏分收集由于具有回收率稳定等优势^［7-9］^，是制备色谱、分离纯化领域中常用的分离手段^［10，11］^。

虽然独立的自动进样器和馏分收集器各自具备上述优点，但目前单一的自动进样器和馏分收集器都只具备独立的功能，无法在自动进样分离和收集功能中实现完全的自动化操作^［12］^，仍存在效率低、需要人工值守、收集方法单一等问题。如Dekker 等^［13］^使用超高效液相色谱仪搭配馏分收集器收集多硫化物中的H_2_S；秦秀秀等^［14］^使用手动收集方法通过半制备型高效液相色谱仪分离纯化吴茱萸中吴茱萸碱和吴茱萸次碱；沈保涛等^［15］^使用制备色谱系统制备纯化生长抑素二聚体，按照时间使用手动收集方法对目标物进行收集。

为解决上述不足，本文研制了集自动进样与馏分收集为一体的制备型设备，该设备控制精准，具有较好的重复性和可靠性。该设备可避免由于人工操作带来样品交叉污染的风险，且便于操作的同时具备多样本处理能力，可提高整体分析能力，适用于大规模实验。综上，该设备具有跨学科应用潜力，可广泛应用于化学、药物研发、环境监测等多个领域，具有较强的通用性。

## 1 设计路线

本文研制了集自动进样与馏分收集为一体的制备型设备（以下简称自动进馏器），其结构设计包括自动进样部分和馏分收集部分。自动进样部分主要完成进样动作，通过软件设置进样方法，仪器接收到进样指令完成自动进样动作；馏分收集部分，则通过软件设置馏分收集方法，仪器通过接收收集指令完成收集动作，实现馏分的自动收集。

本设备的技术路线如图1所示，进样部分和收集部分分别由硬件部分和软件部分组成，硬件部分主要是机械结构设计、电路控制设计；软件部分主要是通过上位机软件实现对进样方法、收集方法、清洗方式等的控制。

**图1:**
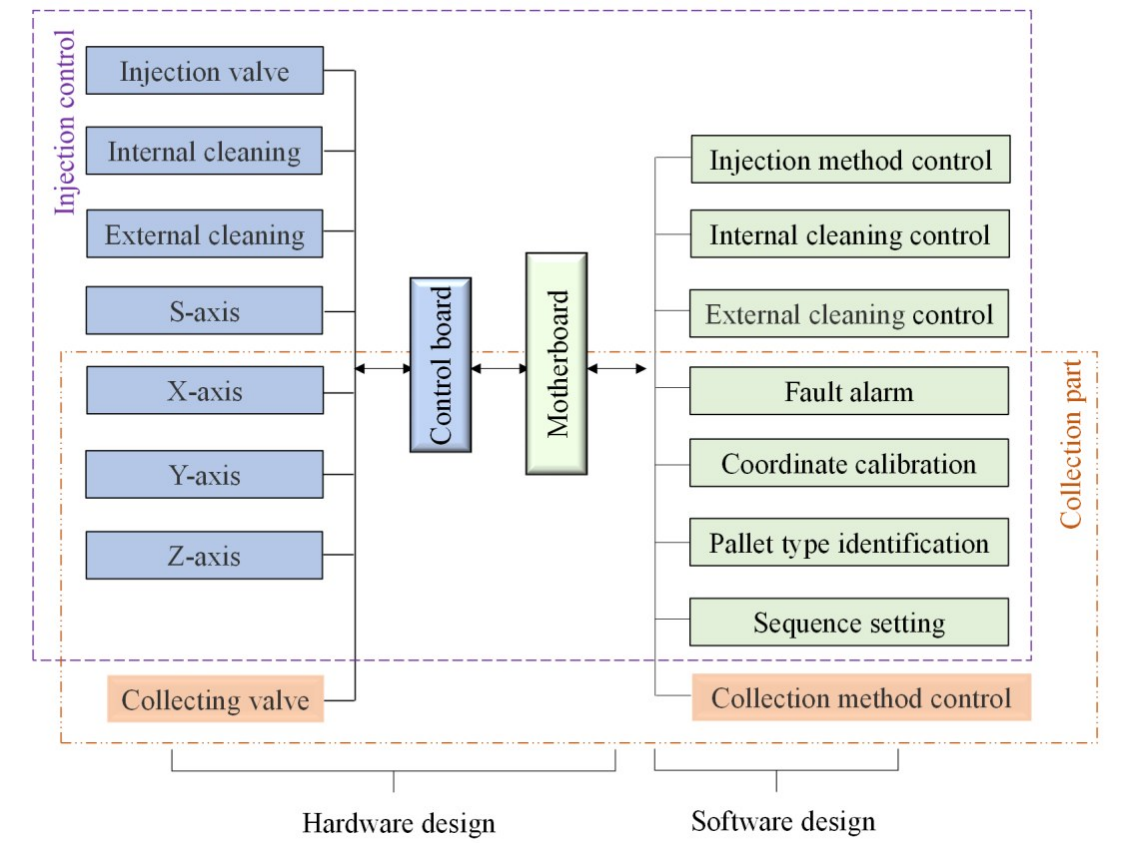
设计路线图

## 2 机械结构设计

本设备的机械设计主要包括X轴、Y轴、Z轴、S轴、进样阀、收集阀、清洗体、蠕动泵等机械组件，这些机械组件均固定在托盘底板上，本设备的机械结构原理图见图2。图2在图1的基础上介绍了各部分机械结构的相对位置和连接关系，以及电路部分对各结构的控制关系。

**图2:**
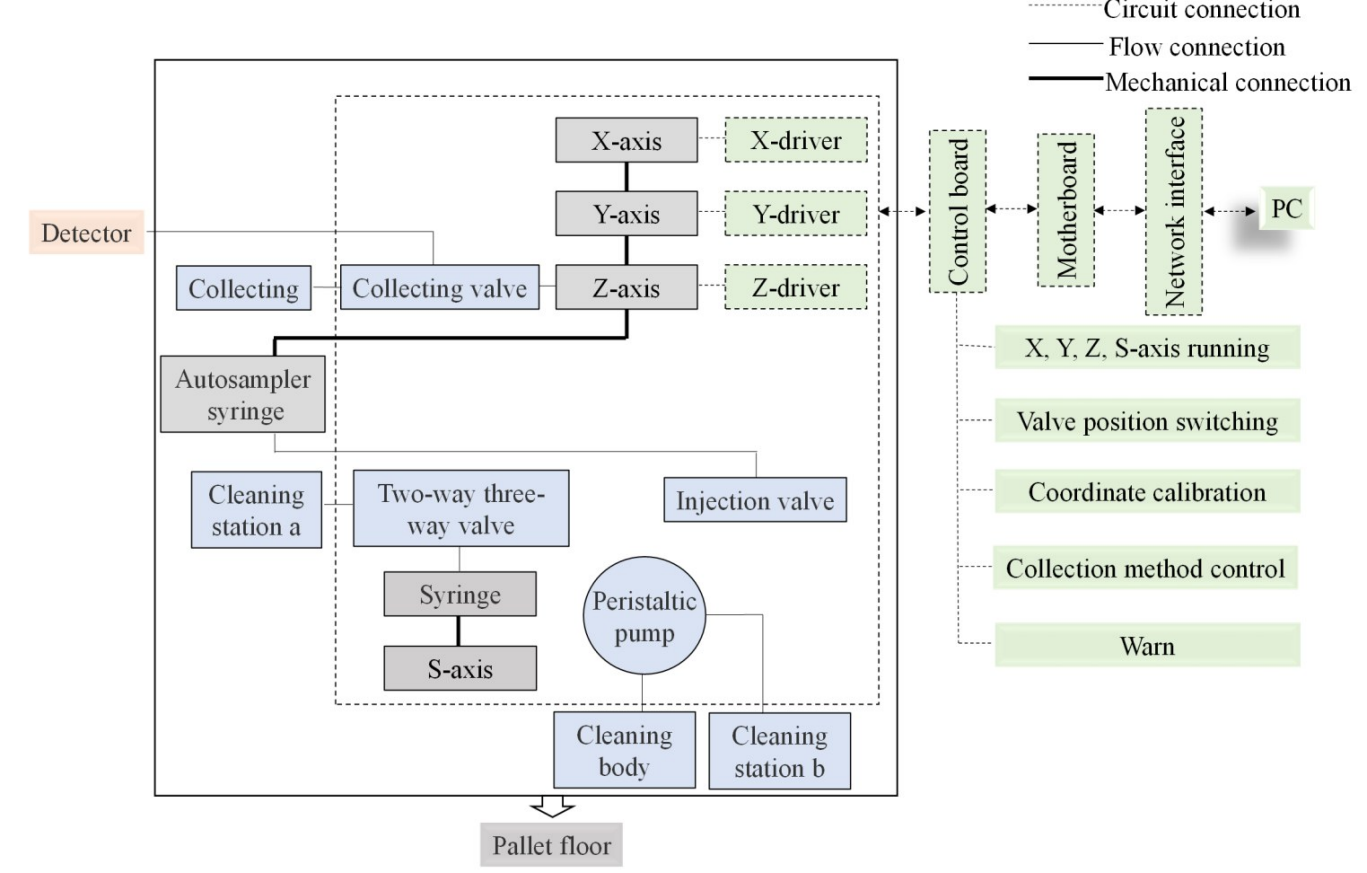
设计原理图

### 2.1 托盘底板

本设备的托盘底板做了独特的设计：一是在托盘四周设置导液槽（如图3的导液槽a、b、c、d），当出现漏液时，液体通过导液槽a（或b、c、d），流经导液槽e，随后进入导液槽f中；二是导液槽f与导液槽e之间存在高度差，因此底盘的液体最终均会流入导液槽f中，最后通过废液口流到废液桶；三是导液槽f中安装漏液传感器，在仪器出现漏液时，漏液传感器将漏液报警信息传递给工作站，工作站弹出漏液报警信息；四是底盘设置有托盘定位销，保证托盘放置在底盘上后不会发生位移，保证了进样和收集托盘位置的准确性；五是底盘设置有4个坐标校准点，在坐标校准时，只需要校准这4个点即可保证任意类型托盘位置的准确性。

**图3:**
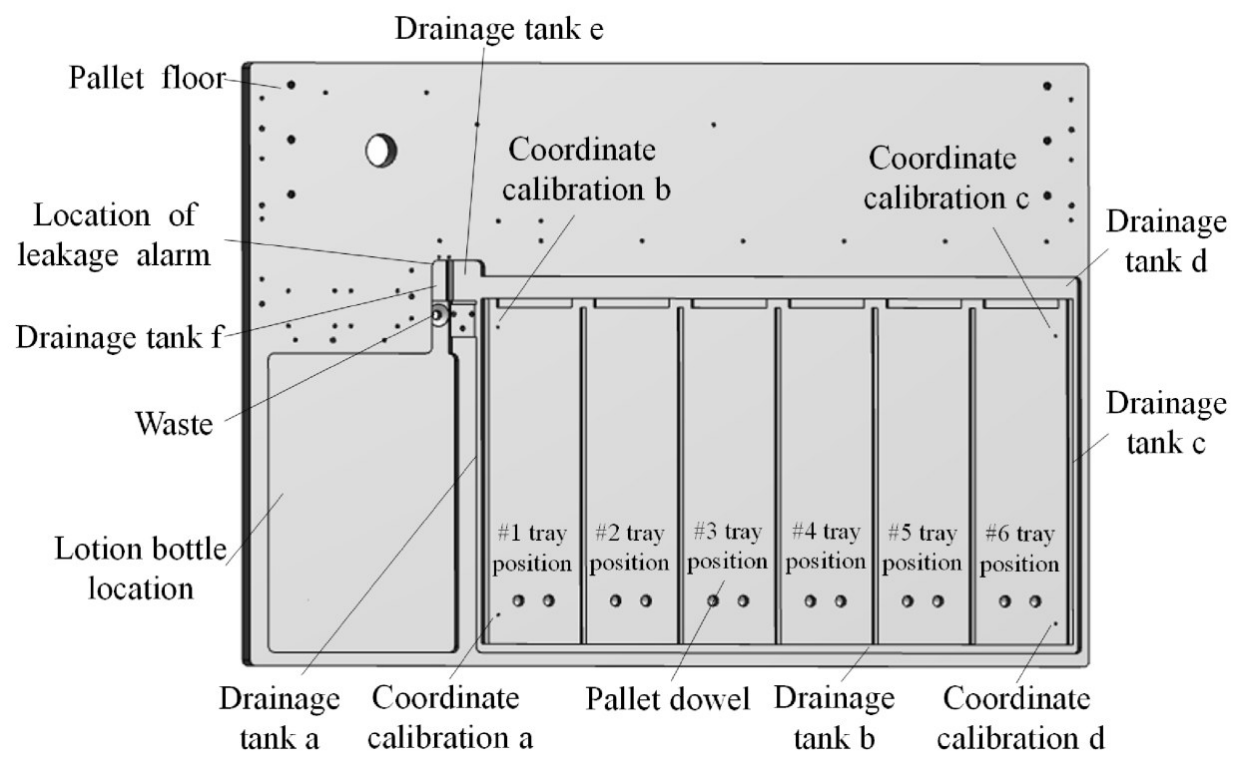
托盘底板示意图

### 2.2 整机

本设备的机械结构采用了开放式设计理念，旨在提供更灵活和便捷的使用体验。进样针通过滑块安装在Z轴上，通过X、Y、Z三维轴运行，实现在图4任意托盘内完成进样动作；收集阀通过固定块安装在Z轴底部，同样通过X、Y、Z三维轴运行，在图4任意托盘内完成收集动作。而运动轴X轴、Y轴、Z轴均采用高速伺服电机，进样步长可以精确到0.2 mm，从而在达到快速、稳定运行的同时，实现进样和收集定位的准确性。同时为防止样品交叉污染，进样和收集为单独流路设计，保证收集纯度。

**图4:**
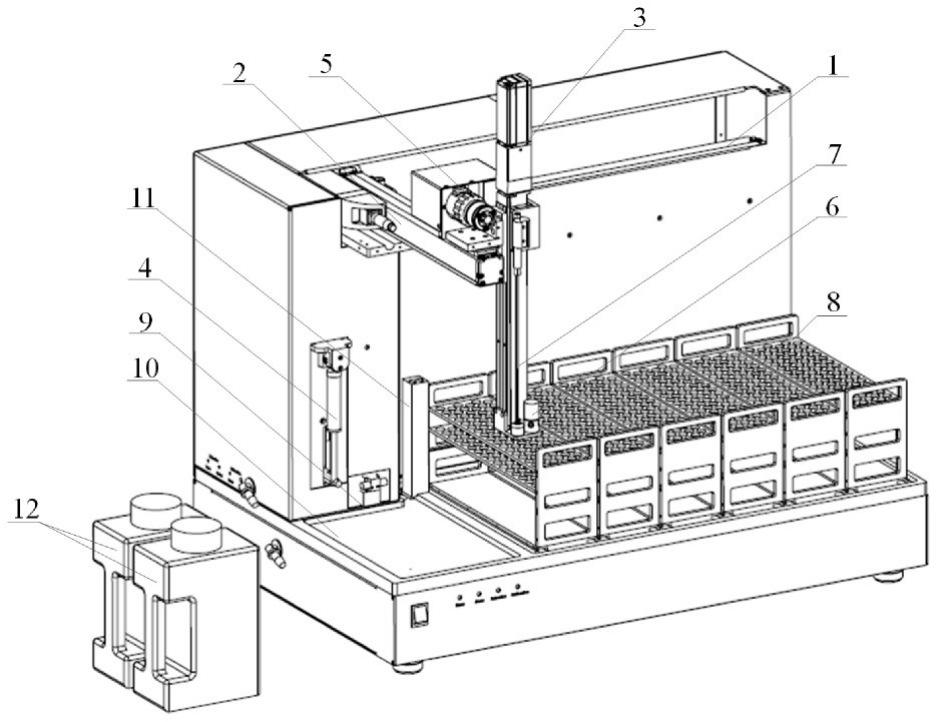
机械结构设计图

## 3 进样和收集方法

### 3.1 坐标算法

为了保证进样和收集位置的准确性，需要对仪器的坐标位置进行校准。同时为了兼容多种规格托盘的任意搭配使用，本文采用了二维坐标系算法。即，以底板为一维坐标系，以X、Y运动轴复位后进样针的位置为一维坐标系的原点建立一维直角坐标系，此时所有托盘都可以用一维坐标系的坐标表示（*x，y*）。再以每个托盘的1号位为二维坐标系原点，分别为每个托盘建立二维直角坐标系（*x_x_，y_y_* ）。在计算过程中先计算每个孔位的二维坐标系的位置，然后与以底板建立的一维坐标系上的位置相加，得到了具体孔位对于机械臂原点的相对位置，即（*x+x_x_，y+y_y_* ）。

本设备的进样和收集动作均在设备底盘范围内完成。底盘设计6个托盘（如图5），且兼容多种规格的样品托盘，根据目前实际使用情况设置了96孔、75孔、70孔、30孔、3孔和双96孔板样品盘（可以满足0.5 mL～1 L液体），每种样品托盘均可以放置在图3和图4底板的任一托盘位置。

**图5:**
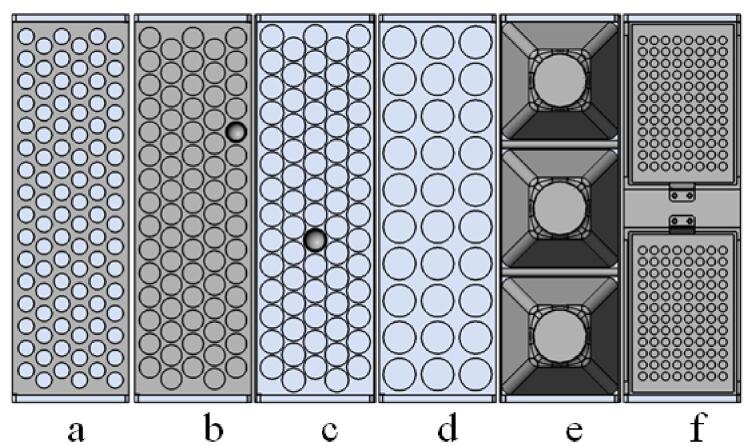
托盘类型

### 3.2 进样方法

该自动进馏器通过工作站控制样品针按X、Y、Z轴3个方向进行三维移动，通过控制注射器和二位六通阀的切换实现进样。

### 3.3 收集方法

该自动进馏器通过工作站采集到样品信号，并根据信号控制收集阀的切换，实现组分的收集。收集阀的切换原理如图6所示，检测器出口连接到COM（common）口。NO（normally open）口用于废液排放，NC（normally closed）口则用于液体收集。在液体收集状态下，工作站会控制收集阀连接至NC口，从而实现液体的收集；在非收集状态时，NO口保持常开，以便于废液的排放。

**图6:**
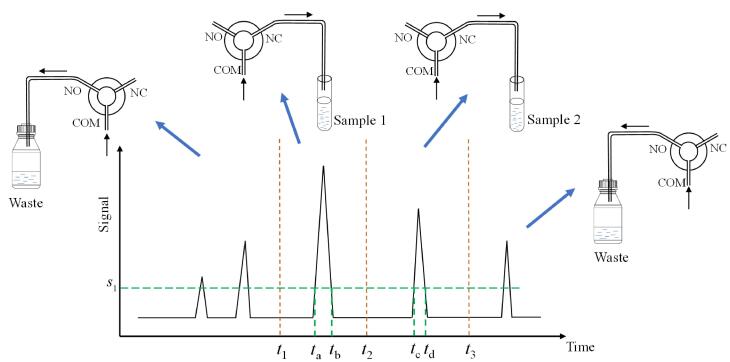
馏分收集的原理

工作站可通过时间、阈值、斜率等参数的运算，控制收集阀的切换位置，达到收集目的。本文以时间、阈值收集方法为例对收集方法进行简单介绍。如图6，通过工作站设置收集方法：*t*_1_到*t*_2_时间内收集样品1，*t*_2_到*t*_3_时间内收集样品2，并且阈值均设置为*s*_1_。实际判断收集动作如下：达到*t*_1_时间后开始判断峰高是否达到*s*_1_，如峰高达到*s*_1_则开始收集样品1，否则不开始收集，即时间*t*和阈值*s*同时满足，则开始收集动作。综上，根据本时间、阈值的方法设置，实际收集动作如下： *t*_a_到*t*_b_时间进行收集，即收集阀的COM口和NC口相通，将样品1收集到指定试管内； *t*_c_到*t*_d_时间进行收集，即收集阀的COM口和NC口相通，并将样品2收集到指定试管内；其余时间COM口与NO口相连，其间的液体作为废液流入废液口。

## 4 设备的性能评价

### 4.1 进样性能评价

参考国家标准GB/T 38125-2019《液相色谱仪用自动进样器》，使用分析天平，进样纯净水，通过称量进样前后纯净水的质量差评价进样器进样准确性。通过试验，得到本文设备进样功能1、2和10 mL的进样误差较小，均在±1.00%范围内（见表1）。

**表1:** 进样误差

Injection volume/mL	Actual volume/mL	Sampling error/%
1	1.0009	0.56
2	2.0066	0.33
10	9.9363	-0.64

### 4.2 样品的制备评价

为了评价该设备对实际样品的制备纯化效果，参考文献［^16^］中对甜菊糖苷的制备方法，使用本文研制的设备对已知含量的甜菊苷（stevioside）和瑞鲍迪苷A（rebaudioside A，RA）样品进行制备。首先使用本设备的进样功能进样10 mL的甜菊糖苷样品，使用本设备的时间和阈值方法对已知含量的甜菊苷和莱鲍迪苷A样品进行收集，并得到甜菊苷和RA纯品。通过对得到的甜菊苷和RA纯品进行液相色谱分析，计算得到甜菊苷和RA的纯度高于93%（见表2）。本文的制备方法，相对文献［^16^］提到的使用三步树脂法工艺得到的甜菊糖苷纯度92.9%略高，结果表明本设备具有较高的分离效率和制备纯度。此外，设备的自动化操作大大缩短了实验周期并提高了操作效率。与文献［^16^］人工操作相比，本设备能够更准确地控制进样量和收集时间，从而提高分离效果和产品质量。同时，设备的兼容性也较好，能够适配多种规格的样品托盘和色谱柱。在流量设计方面，设计流量范围1~200 mL/min，可满足大多数半制备级别的流量需求。

**表2:** 甜菊苷和RA制备纯化结果

Compound	Contents/（mg/mL）		Purity/%
	Actual	Test	
Stevioside	1.00	0.99	95.32
RA	0.96	0.95	93.38

### 4.3 可靠性测试

为了考察该设备在长时间运行的情况下，对进样重复性和收集位置的影响，进行了平均无故障工作时间（mean time between failure，MTBF）为3 000 h的可靠性验证。设置进样和收集方法，在设备连续运行3 000 h后，考察进样准确性和收集位置的准确性。结果如表3所示，该设备在运行3000 h后，连续取样10 mL水样7次，取样误差小于±1%，收集位置准确，未发生偏移，证明该设备可靠性良好。

**表3:** 可靠性评价结果

MTBF/h	Sampling error （*n*=7）/%	Collection location
0	-0.64	accurate
3000	-0.57	accurate

MTBF： mean time between failure.

## 5 结论

本文成功研制了一款全自动进样与馏分收集一体设备，该设备能够实现高效的制备色谱分离操作并具备精确的馏分收集功能。通过实验结果可以看出本设备具有较高的分离效率和制备纯度以及良好的操作性能。未来我们将进一步优化设备的结构和控制系统，以提高其稳定性和可靠性并扩展其应用范围以满足更多领域的制备需求。同时我们还将探索更多新型分离技术的结合以提高分离效率和产品质量并推动相关产业的发展。总之，本文研制的自动进馏器可以满足制药、化工、环境等领域的制备需求，且具有结构简单、便于操作、运行稳定等特点。
